# Serological Profile of Anti-*Toxoplasma gondii* Antibodies in Liver Transplant Recipients

**DOI:** 10.3390/tropicalmed10010018

**Published:** 2025-01-09

**Authors:** Gabriella Beltrame Pintos, Francielly Camilla Bazílio Laurindo Pires, Nathália Zini, Rita Cássia Martins Alves da Silva, Francisco Inaldo Mendes Silva Junior, Renato Ferreira da Silva, Tainara Souza Pinho, Luiz Carlos de Mattos, Cinara Cássia Brandão

**Affiliations:** 1 Faculdade de Medicina de São José do Rio Preto (FAMERP), Avenida Brigadeiro Faria Lima, 5416, Vila São Pedro, São José do Rio Preto 15090-000, SP, Brazilnathalia_zini@hotmail.com (N.Z.); renatosilva@famerp.br (R.F.d.S.); luiz.demattos@famerp.br (L.C.d.M.); 2 Hospital de Base—Fundação Faculdade Regional de Medicina (HB-FUNFARME), São José do Rio Preto 15090-000, SP, Brazil

**Keywords:** solid organ transplantation, toxoplasmosis, transmission routes, screening, chemoprophylaxis

## Abstract

*Toxoplasma gondii (T. gondii)*, a globally distributed obligatory intracellular opportunistic parasite that has infected one third of the world population, has different transmission routes including via organ transplantation. The liver has emerged as a frequent transplanted organ in which the transmission of *T. gondii* can occur between seropositive donors and seronegative recipients. Allied with immunosuppressive therapy, the presence of latent infection in recipients elevates the risk of severe toxoplasmosis. The goal of this study was to evaluate the demographic, clinical, epidemiological, and anti-*T. gondii* antibody profiles in liver transplant recipients. All demographic, clinical, epidemiological, and serological data were obtained from the electronic medical records of liver transplant recipients from the Liver Transplantation Service of the Hospital de Base in São José do Rio Preto, Brazil, from 2008 to 2018. Data from 48 eligible recipients (females: n = 17; males: n = 31) were evaluated. The recipients were grouped according to their *T. gondii* serological profiles (G1: IgM−/IgG−; G2: IgM−/IgG+; G3: IgM+/IgG+; G4: IgM+/IgG−). The overall mean age was 55.3 (±15.3) years; the age difference between women (42.7 ± 17 years) and men (62.2 ± 10.9 years) was statistically significant (*p*-value > 0.0001). The percentages of the serological profiles were 20 (n = 41.7%), 26 (n = 54.1%), and 2 (n = 4.2%) for G1, G2, and G3, respectively. No recipient had a serological profile for G4. Hepatosplenomegaly (47.9%), fever (35.4%), encephalopathy (20.8%), and headache (16.7%) were commonly observed symptoms. No statistically significant differences were observed between the serological group and clinical data (*p*-value = 0.953). The percentages of coinfection by *T. gondii* with hepatitis A, B, and C were 47.9%, 20.8%, and 12.5%, respectively. About 41.7% of the recipients later died. The data demonstrate that infection by *T. gondii* is common in liver transplant recipients, and it is not associated with the analyzed demographic, clinical, and epidemiological data.

## 1. Introduction

Solid organ transplantation (SOT) in humans is a routine procedure in many health services. Although histocompatibility between recipients and donors is mandatory, SOT offers opportunities to investigate a range of infections whose etiological agents can be transmitted through the graft [1]. Despite the high number of publications on SOT, studies focusing on infections transmitted through the graft remain scarce in comparison to other transmission routes such as air, food, and vectors [2].

Among the pathogens transmitted through the graft, *Toxoplasma gondii* stands out. This is an apicomplexan parasite that infects nucleated cells of warm-blooded animals [3]. Its transmission occurs through different routes, such as drinking water contaminated by oocysts or eating raw or undercooked meat and contaminated fruit and vegetables [4,5].

Although *T. gondii* infection remains asymptomatic in most individuals, it can progress to different clinical forms of toxoplasmosis, such as ocular, gestational, and neurological [6]. Clinical toxoplasmosis is rare but can be fatal in immunocompromised patients, causing diseases such as encephalitis, myocarditis, pneumonia, chorioretinitis, and generalized lymphadenopathy [2,7,8,9,10,11,12,13,14,15].

Recipients of organs with latent infection before transplantation are at risk of reactivation of the infection due to immunosuppression [10,13,16,17,18]. Although the first report of toxoplasmosis in a liver graft recipient was published more than five decades ago, the number of studies that evaluate the transmission of *T. gondii* through this medical procedure is still small [16,19,20]. Most reports refer to the transplantation of other organs and tissues, as demonstrated in review articles published within the last decade [9,21,22,23].

As *T. gondii* infects nucleated cells, it can be assumed that the liver is a target organ for this parasite, and its cysts can be transmitted from seropositive donors to seronegative recipients. Therefore, serological screening of donors and recipients before transplantation as well as chemoprophylaxis are mandatory [16].

According to Derouin et al. [7], after organ transplantation, toxoplasmosis continues to be one of the most serious opportunistic infections, and in cases of late diagnosis, the mortality rate is high, occurring in 75% of the patients who did not receive prophylaxis [24]. Webb et al. reported a case of toxoplasmic chorioretinitis in a liver transplant recipient even after prophylaxis, with transmission likely to have occurred through the graft, since the results of serology (a widely used method) occurred late and it was necessary to use molecular methods to confirm infection and diagnosis [17]. In the liver transplant population, toxoplasma infection is a potential risk factor, despite the use of antibiotic prophylaxis with trimethoprim and sulfamethoxazole (TMP/SMX) [25].

Infection by *T. gondii* affects more than one third of the world population; among the most susceptible individuals are solid organ recipients. Since immunosuppression increases the risk of infection and reactivation of toxoplasmosis in these recipients, better knowledge of demographic, clinical, epidemiological, and serological data from recipients with and without *T. gondii* infections will provide the basis for prevention and prophylaxis against morbidity and mortality in the post-transplant period. This study aimed to describe the demographic, clinical, epidemiological, and serological profiles of liver transplant recipients with anti-*T. gondii* antibodies before liver transplant.

## 2. Patients and Methods

### 2.1. Ethics Statement

This retrospective study was approved by the Internal Review Board (IRB) of the Medical school of São José do Rio Preto (FAMERP) on 11 April 2019 under the protocol number CAAE 00726618.6.0000.5415. Because of the nature of the study, a signed consent form was waived.

### 2.2. Study Setting

São José do Rio Preto, located in the northwestern region of the state of São Paulo (SP), Brazil, has a population of 469,173 inhabitants. The municipality has a Human Development Index of 0.834 (https://cidades.ibge.gov.br/brasil/sp/sao-jose-do-rio-preto/panorama accessed on 12 September 2024). The university Hospital de Base from FUNFARME in São José do Rio Preto serves 102 municipalities in the northwestern region of the state of São Paulo and throughout Brazil, through the Brazilian national health service. This hospital has been a reference center for organ and tissue transplants, including liver transplantation, since 1992.

### 2.3. Study Design

This is a descriptive study performed through the analysis of the demographic, clinical, epidemiological, and serological profile of anti-*T. gondii* antibodies, with data on liver transplant recipients from 2008 to 2018 being obtained from the electronic medical records of the Liver Transplantation Service of Hospital de Base in São José do Rio Preto.

The following variables were studied: (1) clinical profile—recipients of liver grafts who presented the following signs and symptoms: (i) fever not justified by any other reason, (ii) lymphadenopathy, (iii) clinical signs of neuroinfection (meningoencephalitis or expansive lesion), (iv) motor deficit related to the central nervous system, (v) abscess without defined cause, (vi) pulmonary nodule, (vii) pneumonitis (diffuse interstitial lesion), (viii) visual alteration (chorioretinitis), (ix) headache without defined etiology, (x) hepatitis (elevation of liver enzymes) without defined etiology, (xi) hepatosplenomegaly without other defined etiology, (xii) encephalopathy, and (xiii) myocarditis; (2) gender; and (3) results of serology for anti-*T. gondii* antibodies (IgM and IgG) using the chemiluminescence test (CLIA, Roche) performed using peripheric blood from cirrhotic patients listed for liver transplantation before the transplant procedure. All manufacturers’ instructions were strictly followed. Data from the organ donors were not included in this study.

### 2.4. Anti-T. gondii Serology

According to the anti-*T. gondii* serology, the liver recipients were divided into four groups: Group 1 (G1): IgM−/IgG−; Group 2 (G2): IgM−/IgG+; Group 3 (G3): IgM+/IgG+; and Group 4 (G4): IgM+/IgG−.

### 2.5. Statistical Analysis

The chi-square test, Fisher’s exact test, and Student’s *t*-test were used to compare the proportions between groups with GraphPad Instat (version 6.3) software. A *p*-value ≤ 5% was considered statistically significant.

## 3. Results

The medical records of 354 liver recipients from 2008 to 2018 were revisited. Only 48 recipients (females: n = 17; males: n = 31) were eligible for analysis. The other recipients were excluded due to missing or incomplete serological data. The overall mean age was 55.3 ± 15.3 years (median: 61.5; range: 21–75 years). The mean age of the male recipients was greater than the age of the females (62.2 ± 10.9 versus 42.7 ± 17 years, respectively; *p*-value > 0.0001).

The serology for anti-*T. gondii* antibodies (Table 1) shows positive results for 28 of the 48 studied patients (58.3%). No recipient had a serological profile for G4. Table 1 also shows the demographic, clinical, and epidemiological data according to the anti-*T*. *gondii* serological profiles.

The number of deaths in G1 was higher for males (n = 7; 77.8%) than for females (n = 2; 22.2%), which was different to G2 (males: n = 5; 55.6% versus females: n = 4; 44.4%), but the differences were not statistically significant (*p*-value = 0.334).

## 4. Discussion

The aim of this study was to report the clinical, epidemiological, and serological profile of anti-*T. gondii* antibodies in liver transplant recipients. The data of this study show that almost two thirds of the recipients studied had antibodies against this apicomplexan parasite, collected on the pre-transplant evaluation, and this signals the need to address attention to the potential possibility of reactivation or de novo infection after transplantation.

Most of the liver recipients presented only IgG anti-*T. gondii* antibodies. The presence of these antibodies characterizes a chronic or previous infection, and there was no evidence of active disease. In our study, some of the clinical characteristics such as fever, headache, hepatosplenomegaly, and encephalopathy were no more significant in recipients with the IgM−/IgG+ profile compared to the IgM−/IgG− profile. Moreover, no association was observed between the serological profile and the occurrence of death.

Infection by *T. gondii* is common in humans, with its prevalence varying according to the region; levels are higher in countries with less socioeconomic development [26,27]. Therefore, it is expected that some liver recipients are chronically infected by this parasite, and we found prevalence similar to that in the literature in our patients.

Our data show that the occurrence of the aforementioned and unspecific clinical signs can be observed even when liver recipients do not present serological evidence of infection by *T. gondii* [24]. Around 4% of the recipients with the IgM−/IgG+ serological profile in this study presented clinical signs. This observation suggests that infection by *T. gondii* in liver recipients can be an important concern, but it is not necessarily associated with clinical signs such as fever, headache, hepatosplenomegaly, or encephalopathy. The investigation for active infection due to *T. gondii* with polymerase chain reaction tests (PCR) could help in the diagnosis of unexplained fever after liver transplantation, especially in the absence of trimethoprim/sulfamethoxazole (TMP/SMX) prophylaxis [23,25].

Our data show that post-transplant death was not associated with the serological profile for anti-*T. gondii* antibodies. In fact, death among organ recipients is a medical concern since the complex transplantation procedures are a high risk regarding postoperative complications and the use of immunosuppressors, among other aspects [28].

Additionally, we observed that the coincidental positive antibody of *T. gondii* and hepatotropic virus was more common with hepatitis A compared to hepatitis B and C antibodies, and we did not find any active hepatitis, even though the hepatitis A virus infection has a benign clinical course. Furthermore, vaccination allows a protective immune response based on specific IgM and IgG antibodies which can be detected during screening for infectious diseases [29,30].

In this study, we did not analyze serology from the liver donors. There is strong evidence that *T. gondii* cysts can be transmitted in the transplanted graft, since a considerable proportion of organ donors have chronic infections with *T. gondii* cysts in their livers. Therefore, it can be assumed that infection by this parasite constitutes an important medical concern for organ recipients [24], and the analysis of transplanted patients in diverse regions can focus the attention to specific population profiles.

Serological investigations of anti-*T. gondii* antibodies in the peripheral blood of organ recipients are carried out by the same methods used for other types of patients. In cases of reagent serology for IgM or IgG anti-*T. gondii* antibodies, chemoprophylaxis is used with the aim of minimizing the risk of reactivation of cysts, whether present in the graft or the recipient, especially due to the immunosuppressive conditions imposed on recipients [7]. We use standard TMP/SMX pneumocystis prophylaxis regimens (TMP 160 mg/SMX 800 mg orally three times weekly or TMP 80 mg/SMX 400 mg orally daily) during the first 3 months for all patients, and for 6 months for patients with high-risk (D+/R−) anti-*T. gondii* antibody profiles. There are reports demonstrating that the reactivation of *T. gondii* infection varies between one week and nine months after the start of immunosuppression and that this condition increases the likelihood of the death of some recipients [31,32,33,34,35].

Transplantation is the first-choice treatment for liver failure patients, providing a good quality of life and with a one-year overall survival rate after transplantation of around 85%. However, infections in the postoperative period are a common cause of morbidity and mortality in these patients. There are reports that demonstrate mortality rates of 26% in the postoperative period [13,15,24], with 88% of these cases being related to infections [36]. Despite the importance of these data, the investigation of infections prior to transplantation and research on its influence on morbidity and mortality rates in the post-transplant period are still scarce.

Dhakal and colleagues demonstrated that the high rate of morbidity in liver transplantation is related to toxoplasmosis. These authors argue that screening to identify anti-*T. gondii* antibodies in liver recipients is low-cost and that its routine use would benefit this group of at-risk patients [37].

Studies have shown that the transmission rate of *T. gondii* from seropositive donors to seronegative recipients in liver transplantation is around 20% [38]. This level is lower than that reported in heart transplants (50–75%), but is much higher than that observed in kidney transplants (1%). The use of serological and molecular laboratory methods can improve the diagnosis of active disease, and when associated with chemoprophylaxis and follow-up can contribute to reducing the transmission rates of *T. gondii* from donors to recipients. These strategies favor the early detection of infection as well as protecting against the potential reactivation of tissue cysts present in the grafts [39].

This study demonstrated that our patients with *T. gondii*’s positive serology before liver transplantation did not have post-transplant signs and symptoms of active disease, nor did the association with death after liver transplantation, probably due to the adopted prophylaxis. This retrospective analysis, as a first evaluation of the pre-liver transplant serology for *T. gondii* in our patients, signals the potential benefit of prospective follow-up studies to investigate the prevalence and clinical and laboratorial profile and to optimize a specific prophylaxis regime and the treatment of *T. gondii* infection among patients undergoing liver transplantation in our region.

The data reported by this study need to be interpreted with caution due to potential limitations, such as not having PCR tests for *T. gondii* during some of the study period, and not including the donors’ results for *T. gondii* serology. This study, as a first evaluation about pre-liver transplant serology of *T. gondii* in our patients, signals the potential benefit of prospective studies to investigate *T. gondii* serology prevalence and clinical and laboratorial profile to optimize the follow-up, prophylaxis regimen, and treatment for this infection among patients undergoing liver transplantation in our region.

## 5. Conclusions

The data from this study demonstrate a high prevalence of anti-*T. gondii* antibodies among liver recipients and suggest that screening and a strategy to identify active infection due to this parasite should be optimized in liver transplantation units.

## Figures and Tables

**Table 1:** Clinical and epidemiological data according to serological profile for IgM and IgG anti-*Toxoplasma gondii* antibodies in 48 liver recipients.

Characteristic	Total (n = 48)		G1 (n = 20)		G2 (n = 26)		G3 (n = 2)	
Anti-*T. gondii* serology (%)			IgG−/IgM− 41.7%		IgG+/IgM− 54.1%		IgG+/IgM+ 4.2%	
Gender								
Male	31		14		15		2	
Female	17		6		11		0	
Mean age (SD)	55.3 ± 15.3		57.3 ± 15.0		54.6 ± 14.0		61.0 ± 15.5	
Median (Min–Max)	61.5 (21–75).		65.0 (58–75)		58.5 (22–70)		61 (50–72)	
				
	n	%	n	%	n	%	n	%
Liver recipients	48	100.0	20	41.7	26	54.1	2	4.2
Symptoms *								
Fever	17	35.4	7	41.2	10	58.8	0	0.0
Lung nodule	1	2.1	0	0.0	1	100.0	0	0.0
Headache	8	16.7	3	37.5	5	62.5	0	0.0
Hepatosplenomegaly	23	47.9	9	39.1	14	60.9	0	0.0
Encephalopathy	10	20.8	4	40.0	6	60.0	0	0.0
Hepatitis *,#								
A	39	81.2	16	41.0	22	56.4	1	2.6
B	15	31.2	5	33.3	9	60.0	1	6.7
C	9	18.7	3	33.3	6	66.7	0	0.0
Coinfections *								
Hepatitis A	23	47.9	0	0.0	22	59.5	1	50.0
Hepatitis B	10	20.8	0	0.0	9	24.3	1	50.0
Hepatitis C	6	12.5	0	0.0	6	16.2	0	0.0
Death *								
Yes	20	41.7	9	45.0	9	34.6	2	100.0
No	28	58.3	11	55.0	17	65.4	0	0.0

SD: Standard deviation; Min: minimum; Max: maximum, *: no statistically significant differences were observed between the variables of G1 compared to G2 + G3; # negative PCR/positive serology.

## Data Availability

No additional data. The data that support the findings of this study are available from the corresponding author upon reasonable request.
